# IN-PERSON FIDELITY MONITORING: HOME-BASED INTERVENTION DELIVERY FOR PERSONS WITH DEMENTIA AND THEIR CAREGIVERS

**DOI:** 10.1093/geroni/igz038.109

**Published:** 2019-11-08

**Authors:** Justine S Sefcik, Nancy A Hodgson

**Affiliations:** University of Pennsylvania, School of Nursing, Philadelphia, Pennsylvania, United States

## Abstract

The gold standard for ensuring satisfactory delivery of an intervention remains fidelity monitoring. In-person observations for fidelity monitoring has received less attention in the literature compared to evaluating sessions via video and audio-recordings. This presentation focuses on a qualitative analysis of field notes from 15 scheduled fidelity visits of a home-based intervention involving persons with dementia and their caregivers (Healthy Patterns, NCT03682185). The findings of advantages (e.g., being able to complete observations in real time), disadvantages (e.g., additional time needed from staff to coordinate and attend visits), and ethical considerations (e.g., potential intrusiveness in the home when observing staff) with be discussed. Future research recommendation is to explore participant perspectives on having an in-person fidelity monitor in the home.

